# The Impact of Motor Symptom Asymmetry on the Relationship Between Non-Motor Manifestations and Neurometabolic Profiles in Parkinson’s Disease

**DOI:** 10.3390/ijms27052120

**Published:** 2026-02-25

**Authors:** Lilla Bonanno, Giulia Marafioti, Alessia Biondo, Amelia Brigandì, Fabrizia Caminiti, Rosa Morabito, Angelo Quartarone, Chiara Sorbera, Rosaria Torre, Rosella Ciurleo

**Affiliations:** IRCCS Centro Neurolesi “Bonino-Pulejo”, Via Palermo S.S. 113, Contrada Casazza, 98124 Messina, Italy; lilla.bonanno@irccsme.it (L.B.); giulia.marafioti@irccsme.it (G.M.); alessia.biondo@irccsme.it (A.B.); amelia.brigandi@irccsme.it (A.B.); fabrizia.caminiti@irccsme.it (F.C.); rosa.morabito@irccsme.it (R.M.); angelo.quartarone@irccsme.it (A.Q.); chiara.sorbera@irccsme.it (C.S.); rosaria.torre@irccsme.it (R.T.)

**Keywords:** globus pallidus, motor symptom asymmetry, non-motor symptoms, neurometabolite, Parkinson’s disease, proton magnetic resonance spectroscopy, substantia nigra

## Abstract

Parkinson’s disease (PD) is characterized by asymmetric motor symptoms (MSs), which may influence non-motor symptoms (NMSs). This study investigated the relationship between NMSs and the neurometabolic profile of the substantia nigra (SN) and globus pallidus (GP) of patients with PD, examining how these associations vary according to MS asymmetry. Forty-three PD patients (20 with right-predominant motor symptoms—RPD, and 23 with left-predominant motor symptoms—LPD) and 20 healthy controls (HCs) underwent single-voxel proton magnetic resonance spectroscopy, along with comprehensive clinical assessments of MSs and NMSs. Compared with HCs, PD patients showed higher N-acetylaspartate (NAA) levels in the SN, lower myo-inositol (Ins) levels in both sides of the SN, and higher glutamate/glutamine (Glx) levels in the right GP. Choline (Cho) in the left GP was positively associated with cognitive performance. In LPD patients, compared with HCs, NAA levels were increased in the right SN, whereas Ins levels were reduced in both hemispheres. These patients reported higher anxiety and exhibited marked hemispheric asymmetry of SN NAA. In this group, higher NAA levels in the right SN were associated with fewer sleep disturbances, while Ins in the right GP was related to both cognitive function and NMS severity. RPD patients showed elevated Glx levels in the right GP compared with HCs, with no significant hemispheric differences in metabolite levels. Nevertheless, Cho in the right SN was positively associated with sleep disturbances. Overall, these findings suggest that motor asymmetry in PD influences the neurometabolic correlates of NMSs, revealing distinct metabolic-clinical profiles in RPD and LPD patients.

## 1. Introduction

Parkinson’s disease (PD) is a progressive neurodegenerative disorder primarily characterized by motor symptoms (MSs), including bradykinesia, rigidity, postural instability, gait disturbances, and resting tremor [1]. These motor manifestations often present asymmetrically, with one side of the body being affected earlier or more severely than the other; however, they typically progress to involve both sides as the disease advances [2,3]. Imaging studies have shown that the asymmetry in PD is generally associated with contralateral neurodegenerative patterns in the basal ganglia [4,5,6,7]. While motor dysfunction remains the clinical hallmark of PD, growing attention has focused on the non-motor symptoms (NMSs) that accompany the disease. The NMSs encompass a wide spectrum of clinical manifestations such as cognitive decline, mood disturbances (depression and anxiety), autonomic dysfunction (orthostatic hypotension, constipation), sleep disorders, and sensory abnormalities [8]. NMSs often precede motor signs and are known to significantly affect patients’ quality of life and overall disease burden [9]. Despite their importance, the pathophysiological mechanisms underlying NMSs in PD remain poorly understood.

Several studies have suggested that the asymmetry of MSs in PD may affect the clinical presentation of NMSs. Specifically, individuals with right-sided MSs, indicating left-hemisphere brain involvement, tend to exhibit greater overall cognitive decline and an increased risk of developing dementia. In contrast, those with left-sided MSs, associated with right-hemisphere pathology, are more likely to experience psychiatric symptoms such as depression and anxiety [10,11,12], or disturbances in sleep [13].

Recent advances in neuroimaging, particularly proton magnetic resonance spectroscopy (^1^H-MRS), have allowed researchers to investigate the neurometabolic profile of the brain in vivo [14]. At 3 Tesla (3T), ^1^H-MRS can detect several primary metabolites, including total N-acetylaspartate + N-acetylaspartylglutamate (NAA), total creatine + phosphocreatine (Cr), total glycerophosphocholine + phosphocholine (Cho), myo-inositol (Ins), and glutamate + glutamine (Glx). NAA is a marker of neuronal health; its reduction indicates neuronal impairment or loss and decreased metabolic activity [15]. Cho reflects membrane synthesis and turnover [16]. Ins is associated with glial cell activity [17]. Glutamate is the main excitatory neurotransmitter in the brain, while glutamine serves as its primary precursor. Cr helps buffer and shuttle energy within cells and is often used as a reference because of its stability across different conditions. Ratios like NAA/Cr, Cho/Cr, Ins/Cr, and Glx/Cr are used as markers for neural density, membrane turnover, gliosis, and neurotransmission, respectively.

An increasing number of studies have reported notable changes in the concentrations of neurometabolites, such as NAA, Cho, Cr, Glx, Ins, and gamma-aminobutyric acid (GABA), in specific brain regions of patients with PD compared to healthy controls (HCs) [18,19,20,21,22,23,24,25]. In particular, lower NAA levels have been found in the substantia nigra (SN), globus pallidus (GP) [20,23], prefrontal lobe, hippocampus, cuneus gyrus, and dorsal thalamus [20] of PD patients compared to HCs. Other studies have reported low Glu levels in the SN [18] and cerebral cortex of PD patients [21]. In addition, higher glutamine levels compared to HCs, and higher glutamate and glutamine levels compared to the contralateral side, were found in the putamen ipsilateral to the more affected hemibody in de novo PD patients [24]. The relative ratios of NAA/Cr, NAA/Cho, and NAA/(Cho + Cr) in the contralateral SN of the affected side were found to be lower than those in the ipsilateral SN in patients with asymmetric PD [19]. Additionally, changes in NAA levels have been reported across all brain lobes, showing clear lateralization contralateral to the predominant symptoms in early-stage PD patients [25].

Some studies have also investigated the relationship between NMSs and neurometabolite concentrations. PD patients with mild cognitive impairment (MCI) showed significantly reduced NAA levels in the SN, GP, prefrontal lobe, hippocampus, cuneus gyrus, and dorsal thalamus [20], as well as reduced NAA, tCr, and Cho concentrations in the posterior cingulate cortex and thalamus [26] compared to HCs. Another study identified alterations in neurochemicals such as Cr, Cho, and Ins as promising biomarkers for predicting cognitive decline in PD. Additionally, Cho/Cr ratios were higher in PD patients with sleep disorders, whereas Ins/Cr ratios were lower in PD patients with gastrointestinal dysfunction compared to those without [27].

Given the asymmetrical presentation of MSs in PD, it is plausible that neurometabolic alterations and their relationship to NMSs may also differ depending on the side of predominant motor involvement. To our knowledge, no study has yet investigated the association between the metabolic profiles of the SN and GP, key regions involved in motor system dysfunction, and the presence of NMSs in PD patients exhibiting lateralized MSs. This represents a gap in the current understanding of the neurochemical basis of symptom heterogeneity in PD.

This study aims to systematically explore the relationship between the neurometabolic profile of SN and GP in PD patients and NMSs, with a specific focus on the role of MS asymmetry. Understanding how MS asymmetry influences the association between NMSs and the neurometabolic profile could shed light on the heterogeneous nature of PD and the underlying neurobiological mechanisms.

## 2. Results

### 2.1. Comparison of Neurometabolite Levels in PD vs. HCs

**Figure 1:**
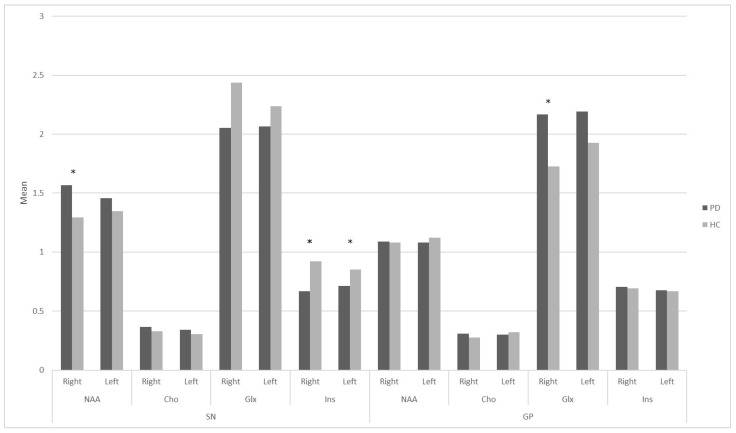
Metabolite concentrations in the substantia nigra and globus pallidus in the two groups. Asterisks indicate significant PD vs. HC differences (*p* < 0.05). Cho = choline; GP = globus pallidus; Glx = combined glutamate and glutamine; HCs = healthy controls; Ins = myo-inositol; NAA = N-acetylaspartate; PD = Parkinson’s disease; SN = substantia nigra.

In the PD vs. HC comparison (PD *n* = 43; HC *n* = 20), several metabolite differences emerged at the nominal level, but none survived multiple-comparison correction. PD patients and HCs did not differ significantly in age (*p* = 0.62) or sex distribution (*p* = 0.72), indicating that the groups were demographically well matched. As expected, however, PD patients showed markedly lower global cognitive performance than HCs on both the MoCA and the MMSE (both *p* < 0.001). Moreover, compared to HCs, PD patients showed higher NAA levels in the right SN (*p* = 0.045; Cohen’s d = 0.50), lower Ins levels in the left (*p* = 0.013; r = −0.31) and in the right SN (*p* = 0.032; Cohen’s d = −0.66), and higher Glx levels in the right GP (*p* = 0.005; r = 0.35) (Figure 1). However, after FDR correction, none of these differences remained statistically significant.

Within the PD group, we found no evidence of hemispheric asymmetry for metabolite levels in the SN or in the GP.

**Figure 2:**
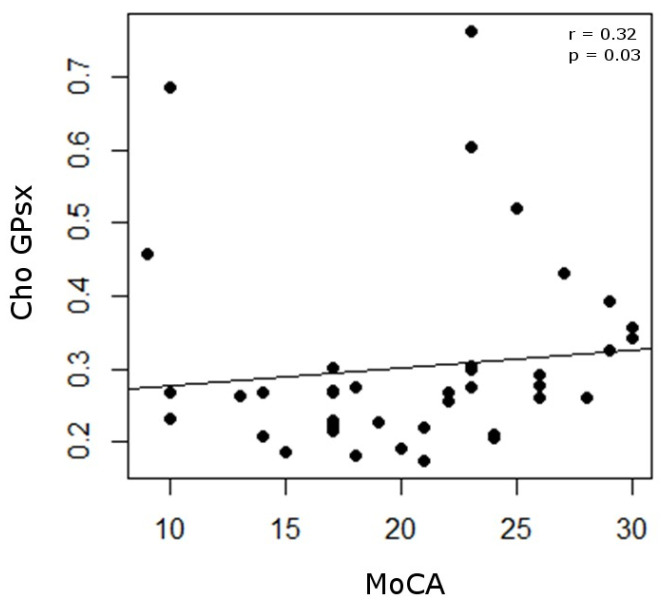
Correlation analysis. For left globus pallidus metabolites, a positive correlation was found between the concentration of Cho and the MoCA score. Cho = choline; GPsx = left globus pallidus; MoCA = Montreal Cognitive Assessment.

Correlation analyses showed positive associations between MoCA score and Cho level in the left GP (r = 0.32, *p* = 0.03) (Figure 2).

### 2.2. Between-Group Analysis

Analysis of metabolite levels across the three groups (RPD, LPD, and HCs) revealed several significant differences. Specifically, for NAA in the right SN, the overall comparison among the three groups did not reach statistical significance (ANOVA, *p* = 0.10) (Table 1). However, NAA in the right SN was significantly increased in the LPD group compared to HCs (*p* = 0.04), with a medium-to-large effect size (d = 0.63) (Table 1). In the same region, Ins levels were also significantly lower in the LPD group compared to HCs (*p* = 0.037), with a smaller effect size (r = 0.32) (Table 1). For Ins in the left SN, comparison across the three groups revealed a significant difference (Kruskal-Wallis, *p* = 0.04) (Table 1). Post hoc analyses indicated that Ins in the left SN was lower in the LPD group compared to HCs (*p* = 0.02, r = 0.35) (Table 1). However, significance did not hold for either comparison after correction for multiple testing.

In the right GP, Glx levels were significantly different among groups (*p* = 0.02) (Table 1), with a marked increase in the RPD group compared to HCs (*p* = 0.01; *p*_adjusted = 0.04; r = 0.41) (Table 1). Comparison between the LPD group and HCs also showed a difference (*p* = 0.02), though it did not survive correction (*p*_adjusted = 0.10) (Table 1).

Cognitive scores also differed significantly among the groups (Table 2). For the MMSE score, significant differences were observed both between the RPD and HC groups (*p* = 0.0005; *p*_adjusted = 0.0021; r = 0.55) and between the LPD and HC groups (*p* = 0.0003; *p*_adjusted = 0.0010; r = 0.56) (Table 2). Similarly, MoCA scores were significantly lower in both PD groups compared to HCs (both *p* < 0.001; *p*_adjusted = 0.0001), with large effect sizes (RPD: r = 0.69; LPD: r = 0.65) (Table 2).

Direct comparison between the two PD groups (RPD vs. LPD) of demographic and clinical variables (age, education, disease duration (DD), and MoCA, MMSE, H&Y, UPDRS-III, NMSS, RBDSQ, COMPASS-31, HARS, and HDRS scores) showed a significant difference only in the HARS scale, with higher scores in the LPD group (Mann-Whitney U, *p* = 0.01; r = 0.37) (Table 2).

### 2.3. Intra-Group Analysis in RPD

**Figure 3:**
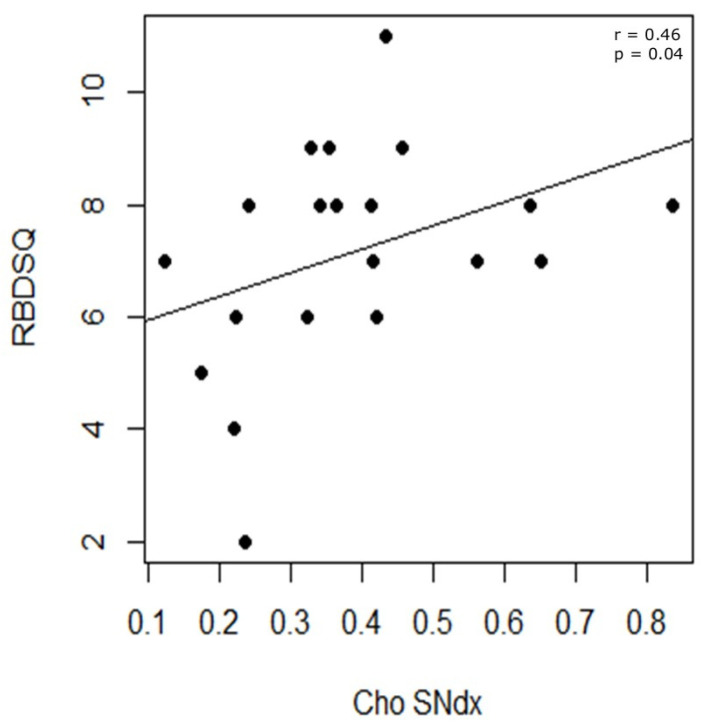
Correlation analysis in RPD patients. For right substantia nigra metabolites, a positive correlation was found between the concentration of Cho and the RBDSQ score. Cho = choline; RBDSQ = REM Sleep Behavior Disorder Screening Questionnaire; SNdx = right substantia nigra.

No significant differences were found between the right and left hemispheres in metabolite levels (NAA, Cho, Glx, Ins) within the SN and GP (Table 1). However, Spearman correlation analyses between metabolite concentrations and clinical variables revealed that Cho levels in the right SN region positively correlated with RBDSQ scores (r = 0.46, *p* = 0.04) (Figure 3).

### 2.4. Intra-Group Analysis in LPD

**Figure 4:**
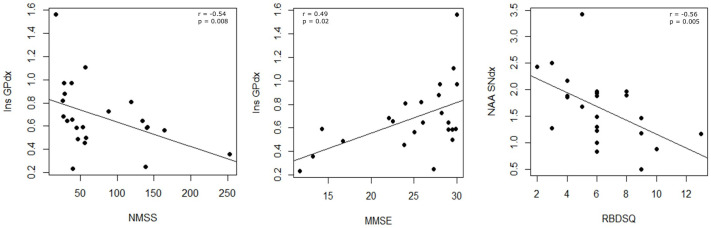
Correlation analysis in LPD patients. For right globus pallidus metabolites, a negative correlation was found between the concentration of Ins and NMSS, and a positive correlation was found between the concentration of Ins and MMSE. For right substantia nigra metabolites, a negative correlation was found between the concentration of NAA and the RBDSQ score. GPdx = right globus pallidus; Ins = myo-inositol; MMSE = Mini Mental State Examination; NAA = N-acetylaspartate; NMSS = Non-Motor Symptoms Scale; RBDSQ = REM Sleep Behavior Disorder Screening Questionnaire; SNdx = right substantia nigra.

The intra-group analysis revealed significant hemispheric asymmetry in NAA levels within the SN of the LPD group (paired *t*-test, *p* = 0.03; d = 0.38) (Table 1), with higher concentrations in contralateral area. Spearman correlations between metabolite levels and clinical variables identified several significant associations. Specifically, Ins levels in the right GP were significantly negatively correlated with NMSS scores (r = −0.54, *p* = 0.008) and positively correlated with MMSE scores (r = 0.49, *p* = 0.02). Moreover, NAA levels in the right SN were negatively correlated with RBDSQ scores (r = −0.56, *p* = 0.005) (Figure 4).

## 3. Discussion

This study investigated the impact of MS asymmetry on the relationship between non-motor manifestations and neurometabolic alterations in PD, with a specific focus on the SN and GP, two regions critically involved in the pathophysiology of motor dysfunction. Moreover, these areas form neural networks that can influence not only motor control, but also cognitive and emotional functions [28,29].

In the overall comparison between PD patients and HCs, several nominal differences in metabolites emerged from ^1^H-MRS analysis, although none survived correction for multiple comparisons. Specifically, PD patients showed higher levels of NAA in the right SN, reduced Ins in the right and left SN, and increased Glx levels in the right GP. The observed increase in NAA in the right SN is an atypical finding, given that most of the literature documents decreased NAA as an indicator of neuronal loss in PD [19,20,23,25]. However, the observed increase in NAA in this region could reflect a compensatory or adaptive metabolic response within surviving neurons, possibly reflecting heightened neuronal activity or altered metabolism aiming to mitigate dopaminergic deficits. Indeed, some studies have reported region-specific variations, including increased NAA/Cr ratios in certain subregions, with caudal SN levels exceeding those in the rostral SN [30,31].

Both the left and the right SN showed evidence of reduced Ins in the PD group relative to HCs, although the findings did not survive correction for multiple comparisons. Ins is predominantly localized to astrocytes, the principal glial cell type, and is thus commonly interpreted as a glial marker. Reduced Ins could point toward astrocytic dysfunction in PD, complementing evidence that astrocytes are key players in dopaminergic neurodegeneration [32,33].

Significant elevations in Glx levels were observed in the right GP of PD patients relative to HCs. Elevated Glx suggests glutamatergic metabolism dysregulation, consistent with previous evidence implicating excitotoxicity and basal ganglia glutamatergic overactivity in PD pathophysiology [34,35]. Furthermore, astrocytes are recognized for their crucial role in the reuptake of glutamate and its conversion into glutamine through the action of glutamine synthetase, a process that helps prevent excitotoxicity [36]. Therefore, astrocyte dysfunction may impair their regulation of glutamate homeostasis, thereby contributing to glutamate-mediated toxicity in PD [37].

The uncorrected correlation analyses revealed a positive association between MoCA scores and Cho levels in the left GP. Because Cho is generally considered an index of membrane turnover and glial/astrocytic activity [38], higher Cho concentrations may indicate preserved cellular integrity that supports cognitive function in PD. This interpretation aligns with previous findings in PD: for example, an ^1^H-MRS study reported that choline levels are altered in patients with cognitive impairment and that MRS neurochemical profiles vary according to cognitive status [26].

When analyzing metabolite levels across PD subgroups (RPD and LPD) and HCs, more nuanced patterns emerged. For NAA in the right SN, although the overall group comparison was not statistically significant, post hoc analyses revealed a significant increase in NAA in the LPD group compared to HCs, with a medium-to-large effect size. NAA is generally considered a marker of neuronal integrity, and elevated NAA in LPD patients may reflect an asymmetry-related compensatory metabolic process or regional differences in disease progression. The lateralization of this result is consistent with the known asymmetry of PD pathology: patients with left-sided symptoms generally exhibit greater neurodegenerative changes in the contralateral (right) SN. The present finding could therefore represent a subpopulation of PD patients in whom neurochemical compensation or delayed degeneration occurs, possibly depending on the more affected side. Similarly, Ins levels in the right SN were lower in the LPD group compared to HCs, albeit with a smaller effect size, and Ins in the left SN was also reduced in LPD patients, indicating potential glial or astrocytic dysfunction. The lateralization of Ins reductions in LPD mirrors the asymmetry of motor symptoms in PD [39] and could indicate that glial metabolic disturbances parallel asymmetric nigral dopaminergic loss. However, significance was lost after correction for multiple comparisons, highlighting the need for cautious interpretation. In the right GP, Glx levels differed significantly between groups, with a marked increase in the RPD group compared to HCs. The comparison between LPD and HCs showed a similar trend that did not survive correction, suggesting that neurochemical substrates of asymmetry extend beyond dopaminergic pathways.

The lack of statistical significance after FDR correction tempers interpretation of these findings. It suggests that, while there are promising nominal trends in neuronal, glial, and neurotransmitter systems, the current sample size or effect magnitudes may not be robust enough to withstand stringent multiple testing. This underscores the exploratory nature of the study. However, the fact that effect sizes in some comparisons (e.g., NAA increase in LPD vs. HCs) reached medium-to-large magnitude indicates biological relevance and motivates further research. The lateralized patterns (e.g., different metabolite changes in RPD vs. LPD) align with the asymmetry of MS that is common in PD, suggesting that motor asymmetry may influence neurochemical trajectories.

Taken together, these data provide preliminary but compelling evidence for region-specific metabolic remodeling in PD, involving neurons (NAA), glial cells (Ins), and excitatory neurotransmission (Glx). The lateralized alterations in RPD vs. LPD point to asymmetric pathophysiology, which may underlie or result from differences in disease progression, compensation, or vulnerability. From a mechanistic perspective, glial dysfunction (as suggested by Ins changes) may impair neuroprotective functions, such as glutamate clearance or metabolic support, thereby contributing to excitotoxic stress (elevated Glx) and eventually neuronal compromise (reflected in NAA changes). These interactions align with emerging evidence of astrocyte metabolic dysregulation in PD [37].

Both PD groups exhibited significantly reduced MMSE and MoCA scores compared to HCs, with large effect sizes. These findings reinforce the high prevalence of cognitive impairment in PD, even in non-demented patients [40,41]. Cognitive decline in PD is multifactorial, involving dopaminergic, cholinergic, and glutamatergic dysfunction across distributed networks [42]. The convergence of neurochemical abnormalities in SN and GP with significant cognitive differences suggests that subcortical metabolic disturbances contribute to non-motor symptoms. When directly comparing LPD and RPD, no major demographic and motor or non-motor differences emerged, except for significantly higher anxiety (HARS scores) in LPD. This aligns with reports linking left-sided symptom onset with greater risk of affective symptoms [10,11,12,43]. This asymmetry may reflect hemispheric specialization in emotional processing, with the right hemisphere (affected in LPD) playing a dominant role in affect regulation [44].

The intra-group analyses in RPD and LPD patients provide further insight into the lateralized neurochemical underpinnings of PD and their association with both MSs and NMSs. Although group-level metabolite asymmetries were not prominent in RPD, clinically relevant correlations emerged, whereas the LPD group showed clear hemispheric asymmetry in NAA within the SN.

Within the RPD group, metabolite levels did not differ significantly across hemispheres in the SN or GP. However, correlation analyses revealed that Cho in the right SN was positively associated with RBDSQ scores, suggesting a link between altered membrane turnover and REM sleep behavior disorder (RBD). Cho is related to phospholipid metabolism and has been implicated in neuroinflammation [16]. The observed association aligns with evidence that RBD is an early marker of widespread neurodegeneration and neuroinflammatory involvement in PD [45].

By contrast, the LPD group exhibited hemispheric asymmetry in NAA within the SN, with higher levels in the contralateral side relative to motor symptom dominance. This finding may represent a compensatory upregulation of neuronal metabolism in the hemisphere contralateral to the more affected side, consistent with prior evidence of asymmetric metabolic and structural changes in PD [39,46].

Correlation analyses further emphasized the clinical relevance of these metabolic asymmetries. NAA in the right SN was negatively correlated with RBDSQ scores, again implicating nigral neuronal metabolism in RBD pathophysiology. Ins levels in the GP showed a dual association: they were negatively correlated with NMSS scores but positively correlated with MMSE. This suggests that astrocytic integrity may contribute to better global non-motor and cognitive outcomes, echoing evidence that astrocytic support is neuroprotective in PD [32].

Taken together, these intra-group analyses reinforce the notion that local neurochemical changes in basal ganglia structures are tightly linked to specific symptom domains in PD. Importantly, the results highlight that both neuronal (NAA) and glial (Ins, Cho) metabolites contribute not only to motor deficits, but also to non-motor disturbances. The asymmetric findings in LPD further underscore the relevance of hemispheric laterality in shaping PD clinical expression.

## 4. Materials and Methods

### 4.1. Study Subjects

Forty-three diagnosed PD patients and 20 HCs, matched for sex and age, were recruited for this study through the Movement Disorder outpatient clinic and/or the Functional Rehabilitation Department at the IRCCS Centro Neurolesi Bonino Pulejo of Messina, Italy between June 2023 and January 2025.

Inclusion criteria for patients included (i) male or female patients aged between 55 and 75 years old; and (ii) clinical diagnosis of PD according to the United Kingdom Parkinson’s Disease Society Brain Bank criteria. Exclusion criteria included (i) severe postural instability; (ii) evident autonomic insufficiency at disease onset; (iii) parkinsonian syndrome; (iv) other neurological or psychiatric diseases; and (v) absolute contraindication to MR imaging (MRI).

HCs with no neurological or psychological disorders had normal cognition (Mini Mental State Examination—MMSE > 26 and Montreal Cognitive Assessment—MoCA > 23).

All subjects provided written informed consent to participate in the study, in accordance with the Declaration of Helsinki. The Ethical Committee of IRCCS Centro Neurolesi Bonino-Pulejo approved the study protocol (approval No. 43/2023) on 3 May 2023.

### 4.2. Clinical Assessment

Disease stage and severity were measured by Hoehn and Yahr stage (H&Y). Motor severity was assessed by Part III of the Unified Parkinson’s Disease Scale (UPDRS-III). The MMSE and MoCA were used to measure global cognitive function in all subjects. NMSs were assessed using the Non-Motor Symptoms Scale (NMSS), sleep disorders were evaluated with the REM Sleep Behavior Disorder Screening Questionnaire (RBDSQ), autonomic dysfunction was measured by the Composite Autonomic Symptom Score-31 (COMPASS-31), and anxiety and depression were assessed using the Hamilton Anxiety Rating Scale (HARS) and the Hamilton Depression Rating Scale (HDRS), respectively.

Clinical laterality was determined based on the motor sub-scores of the UPDRS-III. Accordingly, patients were classified into two groups: PD patients with predominantly right-sided motor symptoms (RPD, *N* = 20) and PD patients with predominantly left-sided motor symptoms (LPD, *N* = 23).

Table 3 shows the demographic and clinical characteristics of all of the groups.

### 4.3. ^1^H-MRS Acquisition and Analysis

All subjects were examined using an MRI protocol which included combined conventional MRI and ^1^H-MRS examinations of the brain. MRI acquisition was performed using 3T whole-body MRI equipment (Achieva, Philips Medical System, Best, The Netherlands) with a 32-element phased array sensitivity-encoding (SENSE) head coil.

**Figure 5:**
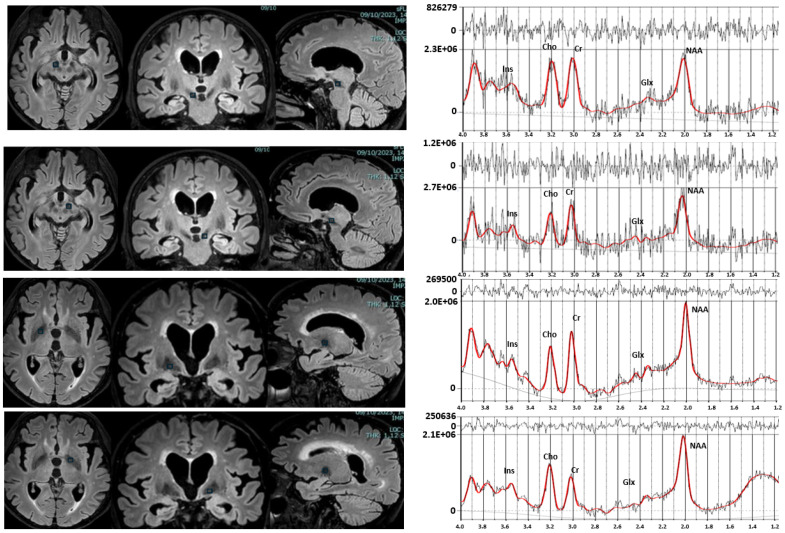
MRI images showing the VOI within the right and left substantia nigra and right and left globus pallidus, along with representative spectra from PD patient. Cho = choline; Cr = creatine; Glx = combined glutamate and glutamine; Ins = myo-inositol; NAA = N-acetylaspartate.

The MR system was equipped with gradients, achieving a maximum slew rate of 200 mT/m/ms and a maximum strength of 80 mT/m. The 3T imaging protocol included 3D T1-weighted fast field echo (FFE), 3D FLAIR, and two-dimensional coronal T2-weighted fast spin echo (FSE). Three-dimensional T1-weighted FFE images were acquired with the following parameters: repetition time (TR) 8.2 ms; echo time (TE) 3.7 ms; section thickness 1 mm; number of signals averaged 1; and reconstruction matrix 512 × 512. 3D-FLAIR images were acquired with TR 12,000 ms and TE 140 ms and were T2-weighted. FSE images were acquired with TR 4100 ms, TE 100 ms, a section of thickness 2–3 mm, number of signals averaged to 1, and a reconstruction matrix of 512 × 512. The MR images were used to select intracranial volumes of interest (VOIs) for spectroscopy that were placed on the GP and SN bilaterally (Figure 5).

For the MRS section, 2D-MRS sequence, point resolved spectroscopy (PRESS) was used (TE: 35 ms, TR: 2000 ms, average 128). The VOI of single-voxel MRS sections was 1.5 cm^3^. Corresponding unsuppressed water spectra with equal TE and TR were additionally acquired. Before data collection, the automatic shim provided by the manufacturer was carried out to optimize field homogeneity.

Metabolite resonance intensities were analyzed using the LCModel/LCMgui software package (Version 6.3; Steven Provencher, Oakville, ON, Canada). Neurometabolite concentrations were estimated by fitting each spectrum to a linear combination of “basic spectra” of each neurometabolite, provided by LCModel software for 3T PRESS acquisition with TE = 35 ms.

Gaussian-fitted peak areas were quantified relative to a baseline calculated from a moving average of noise regions in the spectra. The primary neurometabolites identified included NAA at 2.0 ppm, Cr at 3.0 ppm, Cho at 3.2 ppm, Ins at 3.6 ppm, and a Glx peak at 2.1–2.4 ppm (Figure 5). To ensure data quality, spectra with Cramer–Rao lower bounds exceeding 20% were excluded from the analysis. Neurometabolite levels were expressed as ratios to Cr (NAA/Cr, Cho/Cr, Ins/Cr, Glx/Cr) in the SN and GP. All spectra were acquired and analyzed by an experienced evaluator blinded to participants’ PD status.

### 4.4. Statistical Analysis

Continuous variables were first tested for normality using the Shapiro–Wilk test. We first ran two-group comparisons between the overall PD cohort and HCs using *t*-tests (parametric) or Mann–Whitney U tests (non-parametric), as appropriate. Within the PD group, we then performed intra-subject hemispheric comparisons (right vs. left) for each structure (SN and GP) using paired *t*-tests or Wilcoxon signed-rank tests, depending on distribution. We also explored correlations between neurometabolites and clinical variables using Spearman’s ρ.

Subsequently, the PD sample was divided according to the more affected side (RPD and LPD) and analyzed together with the HCs. Based on the distribution, either one-way ANOVA or the Kruskal–Wallis test was applied to assess group differences among the three groups: RPD, LPD and HCs. For post hoc comparisons, the Tukey HSD test was used following ANOVA, and Dunn’s test with Benjamini–Hochberg correction for multiple comparisons was applied after Kruskal–Wallis. Across families of related tests, multiple comparisons were controlled using either the false discovery rate (FDR) or Bonferroni correction, depending on the specific analysis. Pairwise comparisons between the two PD groups were performed using either paired *t*-tests or Mann–Whitney U tests, depending on normality. For intra-group comparisons between hemispheres (right vs. left), paired *t*-tests or Wilcoxon signed-rank tests were employed. Effect sizes were calculated to assess the magnitude of differences—Cohen’s d for parametric comparisons and r for non-parametric ones—and interpreted according to conventional thresholds (small: 0.1, medium: 0.3, large: 0.5). Correlations between metabolite levels and clinical variables were explored using Spearman’s rank correlation coefficients (ρ). All *p*-values were two-tailed, and statistical significance was set at *p* < 0.05. Adjusted *p*-values are reported where applicable to account for multiple comparisons. The statistical analyses were conducted using R software (version 4.4.2).

## 5. Conclusions

The present study extends the literature by identifying specific neurometabolites in basal ganglia structures as potential intermediate biomarkers mediating the link between lateralized motor involvement and NMSs. Overall, the findings suggest that metabolic alterations in PD are both region- and side-specific, potentially reflecting the asymmetric nature of the disease. While many effects did not remain significant after correction, the observed trends in NAA, Ins, and Glx point to alterations in neuronal integrity, glial function, and excitatory neurotransmission in the SN and GP.

The current literature lacks consensus regarding the underlying causes of MS asymmetry in PD. Increasing experimental and clinical evidence supports the notion that PD may originate in the periphery, particularly within the enteric autonomic system, years before the onset of classical motor manifestations, as proposed in the Braak staging hypothesis [47]. In addition, the α-synuclein origin site and connectome (SOC) model proposed by Borghammer [48] suggests that PD progression depends on both the initial site of α-synuclein pathology and its spread along dominant brain connections. In the “brain-first” subtype, pathology begins unilaterally within the central nervous system (often in the amygdala), spreading mainly to the same hemisphere, leading to asymmetric dopaminergic degeneration and MSs. In the “body-first” subtype, pathology starts in the peripheral autonomic nervous system and spreads bilaterally to the brain via the vagus nerve, resulting in more symmetric brain involvement and less motor asymmetry, but faster progression and earlier cognitive decline. While anatomical differences between the two vagus nerves are recognized, it is not yet established that these differences systematically translate into asymmetric central neurodegeneration [2]. Therefore, asymmetric dopaminergic degeneration within the nigrostriatal system remains the best-characterized substrate of lateralized motor symptoms [4,5,6,7]. Starting from this consideration, our findings reinforce the hypothesis that motor asymmetry may play a role in influencing the relationship between neurobiological substrates and NMS profile in PD.

However, it is possible that early peripheral asymmetry may interact with intrinsic hemispheric vulnerability to shape later motor lateralization. Therefore, the important and still open question of whether asymmetry is already present during the peripheral phase, and whether differences in vagal anatomy contribute to the lateralized central phenotype, warrants further investigation.

Several limitations should be acknowledged, including the relatively small sample size, which reduces statistical power, and the cross-sectional design, which precludes causal inferences or assessment of longitudinal trajectories. These limitations underscore the need for caution in interpreting the findings. In addition, future longitudinal studies with larger cohorts and multimodal integration of spectroscopy, structural and functional imaging, and clinical markers are warranted to clarify whether these neurometabolic alterations represent compensatory mechanisms, markers of disease progression, or epiphenomena of asymmetric pathology.

In conclusion, our results highlight the importance of considering MS asymmetry in the clinical and biological characterization of PD and suggest that neurometabolic profiling of SN and GP may provide valuable insights into the neurobiological mechanisms underlying symptom heterogeneity, paving the way for stratified and personalized therapeutic approaches.

## Figures and Tables

**Table 1:** Inter-and intra-group comparisons of metabolite concentrations in the SN and GP for RPD, LPD, and HCs.

Region	Metabolite	Side	RPD	LPD	HCs	Inter-Group	RPD vs. HCs		LPD vs. HCs		RPD vs. LPD	
			*Mean ± SD*	*Mean ± SD*	*Mean ± SD*	*p*	*p*	*Effect size*	*p*	*Effect size*	*p*	*Effect size*
SN	NAA	Right	1.47 ± 0.45	1.65 ± 0.65	1.29 ± 0.44	0.10 ^¥^	0.22 º	d = 0.40	0.04 *º	d = 0.63	0.31 º	d = −0.31
		Left	1.51 ± 0.47	1.41 ± 0.58	1.35 ± 0.54	0.61 ^¥^	0.30 º	d = 0.33	0.72 º	d = 0.11	0.52 º	d = 0.20
		*p*	0.66 ^±^	0.03 *^±^	0.77 ^±^							
	Cho	Right	0.39 ± 0.18	0.35 ± 0.12	0.33 ± 0.11	0.67 ^+^	0.23 º	d = 0.40	0.67 ^§^	r = 0.06	0.65 ^§^	r = 0.07
		Left	0.34 ± 0.13	0.35 ± 0.13	0.31 ± 0.09	0.62 ^+^	0.54 ^§^	r = 0.10	0.24 º	d = 0.35	0.68 ^§^	r = 0.06
		*p*	0.36 ^±^	0.86 ^±^	0.45 ^±^							
	Glx	Right	2.14 ± 0.78	1.98 ± 0.66	2.44 ± 1.46	0.72 ^+^	0.92 ^§^	r = 0.01	0.39 ^§^	r = 0.13	0.48 º	d = 0.22
		Left	2.16 ± 0.52	1.98 ± 0.89	2.24 ± 0.88	0.27 ^+^	0.75 º	d = −0.10	0.31 ^§^	r = 0.15	0.11 ^§^	r = 0.25
		*p*	0.88 ^±^	0.99 ^±^	0.54 ^±^							
	Ins	Right	0.69 ± 0.31	0.65 ± 0.38	0.92 ± 0.45	0.07 ^+^	0.06 º	d = −0.61	0.04 *^§^	r = 0.32	0.46 ^§^	r = 0.11
		Left	0.72 ± 0.45	0.71 ± 0.59	0.85 ± 0.35	0.04 *^+^	0.05 ^§^	r = 0.32	0.02 *^§^	r = 0.35	0.72 ^§^	r = 0.05
		*p*	0.78 ^±^	0.87 ^χ^	0.52 ^±^							
GP	NAA	Right	1.10 ± 0.34	1.08 ± 0.27	1.08 ± 0.41	0.92 ^+^	0.86 º	d = 0.06	0.66 ^§^	r = 0.07	0.99 ^§^	r = 0.00
		Left	1.00 ± 0.30	1.15 ± 0.44	1.12 ± 0.34	0.15 ^+^	0.13 ^§^	r = 0.24	0.53 ^§^	r = 0.10	0.09 ^§^	r = 0.26
		*p*	0.28 ^±^	0.34 ^χ^	0.68 ^±^							
	Cho	Right	0.29 ± 0.08	0.32 ± 0.15	0.28 ± 0.10	0.59 ^+^	0.30 ^§^	r = 0.16	0.47 ^§^	r = 0.11	0.93 ^§^	r = 0.01
		Left	0.28 ± 0.13	0.32 ± 0.13	0.32 ± 0.10	0.18 ^+^	0.08 ^§^	r = 0.28	0.49 ^§^	r = 0.11	0.22 ^§^	r = 0.19
		*p*	0.44 ^χ^	0.64 ^χ^	0.12 ^±^							
	Glx	Right	2.22 ± 0.66	2.12 ± 0.73	1.73 ± 0.82	0.02 *^+^	0.01 *^§^	r = 0.41	0. 02 *^§^	r = 0.34	0.71 ^§^	r = 0.06
		Left	2.31 ± 1.04	2.09 ± 0.59	1.93 ± 0.84	0.32 ^+^	0.18 ^§^	r = 0.21	0.26 ^§^	r = 0.17	0.42 º	d = 0.26
		*p*	0.56 ^χ^	0.88 ^±^	0.07 ^±^							
	Ins	Right	0.73 ± 0.36	0.68 ± 0.29	0.69 ± 0.30	0.88 ^¥^	0.73 º	d = 0.11	0.91 º	d = −0.04	0.64 º	d = 0.15
		Left	0.66 ± 0.26	0.69 ± 0.45	0.67 ± 0.39	0.88 ^+^	0.91 º	d = −0.04	0.98 ^§^	r = 0.00	0.66 ^§^	r = 0.07
		*p*	0.42 ^±^	0.64 ^χ^	0.81 ^±^							

* *p* < 0.05; ^¥^ ANOVA-one way test; ^+^ Kruskal–Wallis test; ^±^ Paired *t*-test; ^χ^ Wilcoxon signed rank test; º Unpaired *t*-test; ^§^ Mann–Whitney U test. Pairwise comparisons are provided for each group pair, with *p*-values and effect sizes: *r* for non-parametric tests and Cohen’s *d* for parametric tests. The r effect size was calculated as r=ZN, where Z is the standardized test statistic and N is the total number of observations. Legend: Cho = choline; Glx = glutamate + glutamine; GP = globus pallidus; HCs = healthy controls; Ins = myo-inositol; LPD = PD patients with predominantly left-sided motor symptoms; NAA = N-acetylaspartate; RPD = PD patients with predominantly right-sided motor symptoms; SN = substantia nigra.

**Table 2:** Inter-group comparisons for demographic and clinical variables among RPD, LPD, and HCs.

	RPD	LPD	HCs	Inter-Group	RPD vs. HCs		LPD vs. HCs		RPD vs. LPD	
	*Mean ± SD*	*Mean ± SD*	*Mean ± SD*	*p*	*p*	*Effect size*	*p*	*Effect size*	*p*	*Effect size*
Age	64.20 ± 6.25	67.30 ± 8.84	64.95 ± 6.29	0.24 ^+^	0.71	d = −0.12	0.21 ^§^	r = 0.19	0.11 ^§^	r = 0.24
Education	9.05 ± 3.65	9.65 ± 4.34	-	-	-	-	-	-	0.76 ^§^	r = 0.05
DD	8.95 ± 5.68	8.04 ± 4.46	-	-	-	-	-	-	0.68 ^§^	r = 0.06
LEDD	695.5 ± 337.29	624.77 ± 283.40	-	-	-	-	-	-	0.47 º	d = 0.23
MoCA	20.25 ± 5.55	19.96 ± 6.46	27.95 ± 3.03	<0.001 *^+^	<0.001 *^§^	r = 0.69	<0.001 *^§^	r = 0.65	0.87 º	d = 0.05
MMSE	24.12 ± 4.89	24.91 ± 5.72	29.03 ± 1.89	<0.001 *^+^	<0.001 *^§^	r = 0.55	<0.001 *^§^	r = 0.56	0.52 ^§^	r = 0.10
HY	2.53 ± 0.85	2.48 ± 0.50	-	-	-	-	-	-	0.69 ^§^	r = 0.06
UPDRS-III	38.30 ± 13.39	41.96 ± 12.36	-	-	-	-	-	-	0.36 º	d = −0.28
NMSS	66.60 ± 62.04	76.96 ± 59.95	-	-	-	-	-	-	0.54 ^§^	r = 0.09
RBDSQ	7.15 ± 1.98	6.26 ± 2.58	-	-	-	-	-	-	0.21 º	d = 0.38
COMPASS-31	21.17 ± 10.70	31.88 ± 17.93	-	-	-	-	-	-	0.05 ^§^	r = 0.29
HARS	14.20 ± 6.79	19.22 ± 7.77	-	-	-	-	-	-	0.02 *^§^	r = 0.37
HDRS	14.20 ± 7.08	16.78 ± 6.27	-	-	-	-	-	-	0.15 ^§^	r = 0.22

* *p* < 0.05; ^+^ Kruskal–Wallis test; º Unpaired *t*-test; ^§^ Mann–Whitney U test. Pairwise comparisons are provided for each group pair, with *p*-values and effect sizes: r for non-parametric tests and Cohen’s d for parametric tests. The r effect size was calculated as r = Z⁄√N, where Z is the standardized test statistic and N is the total number of observations. Legend: COMPASS-31 = Composite Autonomic Symptom Score 31; DD = disease duration; HARS = Hamilton Anxiety Rating Scale; HCs = healthy controls; HDRS = Hamilton Depression Rating Scale; HY = Hoehn and Yahr scale; LPD = PD patients with predominantly left-sided motor symptoms; MMSE = Mini Mental State Examination; MoCA = Montreal Cognitive Assessment; NMSS = Non-Motor Symptoms Scale; RBDSQ = REM Sleep Behavior Disorder Screening Questionnaire; RPD = PD patients with predominantly right-sided motor symptoms; SD: standard deviation; UPDRS-III = Unified Parkinson’s Disease Rating Scale—Part III.

**Table 3:** Demographics and clinical characteristics of patients with PD, RPD, or LPD and HCs.

	PD (*N* = 43)	RPD (*N* = 20)	LPD (*N* = 23)	HCs (*N* = 20)
Age	65.86 ± 7.82	64.20 ± 6.25	67.30 ± 8.84	64.95 ± 6.29
Education	9.37 ± 4.00	9.05 ± 3.65	9.65 ± 4.34	14.65 ± 4.0
**Gender**				
Male	23	12	11	9
Female	20	8	12	11
DD	8.46 ± 5.02	8.95 ± 5.68	8.04 ± 4.46	-
LEDD	648.90 ± 308.44	695.5 ± 337.29	624.77 ± 283.40	-
MoCA	20.09 ± 5.99	20.25 ± 5.55	19.96 ± 6.46	27.95 ± 3.03
MMSE	24.54 ± 5.30	24.12 ± 4.89	24.91 ± 5.72	29.03 ± 1.89
H&Y	2.52 ± 0.67	2.53 ± 0.85	2.48 ± 0.50	-
UPDRS-III	40.26 ± 12.83	38.30 ± 13.39	41.96 ± 12.36	-
NMSS	72.14 ± 60.42	66.60 ± 62.04	76.96 ± 59.95	-
RBDSQ	6.67 ± 2.34	7.15 ± 1.98	6.26 ± 2.58	-
COMPASS-31	26.87 ± 15.77	21.17 ± 10.70	31.88 ± 17.93	-
HARS	16.88 ± 7.67	14.20 ± 6.79	19.22 ± 7.77	-
HDRS	15.58 ± 6.71	14.20 ± 7.08	16.78 ± 6.27	-

Legend: COMPASS-31 = Composite Autonomic Symptom Score-31; DD = disease duration; HARS = Hamilton Anxiety Rating Scale; HCs = healthy controls; HDRS = Hamilton Depression Rating Scale; H&Y = Hoehn and Yahr scale; LEDD = L-dopa equivalent daily dose; LPD = PD patients with predominantly left-sided motor symptoms; MMSE = Mini Mental State Examination; MoCA = Montreal Cognitive Assessment; NMSS = Non-Motor Symptoms Scale; PD = Parkinson’s disease; RBDSQ = REM Sleep Behavior Disorder Screening Questionnaire; RPD = PD patients with predominantly right-sided motor symptoms; UPDRS-III = Unified Parkinson’s Disease Rating Scale—Part III.

## Data Availability

Data reported in this study are available from the corresponding author on reasonable request. The data supporting the study results are not publicly available, for confidentiality reasons.

## Abbreviations

The following abbreviations are used in this manuscript:

^1^H-MRS	Proton magnetic resonance spectroscopy
Cho	Choline
COMPASS-31	Composite Autonomic Symptom Score-31
Cr	Creatine
DD	Disease duration
GABA	Gamma-aminobutyric acid
Glx	Glutamate + glutamine
GP	Globus pallidus
H&Y	Hoehn and Yahr stage
HARS	Hamilton Anxiety Rating Scale
HCs	Healthy controls
HDRS	Hamilton Depression Rating Scale
Ins	Myo-inositol
LEDD	L-dopa equivalent daily dose
LPD	PD patients with predominantly left-sided motor symptoms
MCI	Mild cognitive impairment
MMSE	Mini Mental State Examination
MoCA	Montreal Cognitive Assessment
MRI	Magnetic Resonance Imaging
MSs	Motor symptoms
NAA	N-acetylaspartate
NMSs	Non-motor symptoms
NMSS	Non-Motor Symptoms Scale
PD	Parkinson’s disease
RBD	REM sleep behavior disorder
RBDSQ	REM Sleep Behavior Disorder Screening Questionnaire
RPD	PD patients with predominantly right-sided motor symptoms
SN	Substantia nigra
TE	Echo time
TR	Repetition time
UPDRS-III	Unified Parkinson’s Disease Scale—Part III
VOI	Volumes of interest
